# A threshold method for immunological correlates of protection

**DOI:** 10.1186/1471-2288-13-29

**Published:** 2013-03-01

**Authors:** Xuan Chen, Fabrice Bailleux, Kamal Desai, Li Qin, Andrew J Dunning

**Affiliations:** 1 Sanofi Pasteur, Beijing, China; 2 Sanofi Pasteur, Marcy L’Etoile, France; 3 United Biosource Corporation, London, UK; 4 Statistical Center for HIV/AIDS Research and Prevention, Fred Hutchinson Cancer Research Center, Seattle, WA, USA; 5 Current address: Amazon.com, Inc, Seattle, WA, USA; 6 Sanofi Pasteur, Swiftwater, PAUSA

**Keywords:** Vaccine, Correlate of protection, Protective threshold, Immunological assay

## Abstract

**Background:**

Immunological correlates of protection are biological markers such as disease-specific antibodies which correlate with protection against disease and which are measurable with immunological assays. It is common in vaccine research and in setting immunization policy to rely on threshold values for the correlate where the accepted threshold differentiates between individuals who are considered to be protected against disease and those who are susceptible. Examples where thresholds are used include development of a new generation 13-valent pneumococcal conjugate vaccine which was required in clinical trials to meet accepted thresholds for the older 7-valent vaccine, and public health decision making on vaccination policy based on long-term maintenance of protective thresholds for Hepatitis A, rubella, measles, Japanese encephalitis and others. Despite widespread use of such thresholds in vaccine policy and research, few statistical approaches have been formally developed which specifically incorporate a threshold parameter in order to estimate the value of the protective threshold from data.

**Methods:**

We propose a 3-parameter statistical model called the a:b model which incorporates parameters for a threshold and constant but different infection probabilities below and above the threshold estimated using profile likelihood or least squares methods. Evaluation of the estimated threshold can be performed by a significance test for the existence of a threshold using a modified likelihood ratio test which follows a chi-squared distribution with 3 degrees of freedom, and confidence intervals for the threshold can be obtained by bootstrapping. The model also permits assessment of relative risk of infection in patients achieving the threshold or not. Goodness-of-fit of the a:b model may be assessed using the Hosmer-Lemeshow approach. The model is applied to 15 datasets from published clinical trials on pertussis, respiratory syncytial virus and varicella.

**Results:**

Highly significant thresholds with p-values less than 0.01 were found for 13 of the 15 datasets. Considerable variability was seen in the widths of confidence intervals. Relative risks indicated around 70% or better protection in 11 datasets and relevance of the estimated threshold to imply strong protection. Goodness-of-fit was generally acceptable.

**Conclusions:**

The a:b model offers a formal statistical method of estimation of thresholds differentiating susceptible from protected individuals which has previously depended on putative statements based on visual inspection of data.

## Background

Immunological correlates of protection are measurable and specific biological markers which correlate with protection against disease caused by an infectious pathogen. The markers used are most often pathogen-specific neutralizing antibodies whose concentration can be measured with biological assays [1]. Researchers and agencies responsible for immunization recommendations, such as the US Advisory Committee for Immunization Practices and the World Health Organization, rely on established threshold values for the immunological correlate of protection where the accepted threshold differentiates between individuals who are considered to be immunologically protected against disease and those who are susceptible [2,3]. When it is strongly correlated with protection with a recognized threshold, it can be called an absolute correlate [4].

Uses for the established threshold for a correlate of protection are numerous. For instance, where the correlate has been established for a vaccine that has already demonstrated clinical efficacy against disease, the correlate simplifies study of the vaccine in new populations, age- or risk-groups by permitting clinical trials to be conducted with immunogenicity endpoints and avoiding large-scale efficacy trials. The US Food and Drug Administration (FDA) offers accelerated approval when there is a correlate (FDA prefers the term “surrogate”) that is considered “reasonably likely” to predict clinical benefits [5]. Other uses include the study of immunogenicity for co-administration with other vaccines, comparisons of combination vaccines to individual component vaccines and assessment of the duration of protection. The established correlate of protection also permits comparisons of new generation vaccines to older ones. For completely novel vaccines, the demonstration of a candidate immunologic correlate is becoming a secondary yet fundamental objective in clinical trials and epidemiological studies. This is encouraged by agencies such as the US FDA Center for Biologics Evaluation and Research and is one of the Grand Challenges in Global Health [6]. Thus the accurate identification of protective threshold levels clearly has important implications for the licensure of vaccines and for immunization policy.

Research in correlates of protection is multidisciplinary. As a consequence, terminology used has been inconsistent and sometimes confusing. There have been recent efforts to harmonize the terminology employed and to link this to a hierarchy of statistical evidence for the demonstration of a correlate [4,7,8]. In addition the terminology has been further refined by introducing the terms mechanistic and nonmechanistic to address whether the correlate of protection is causal or not [9]. We will here for convenience use the term ‘correlate of protection’ in the broadest sense, to include immunological assays that have been consistently shown to correlate with risk of disease, assays that have been shown to be causally associated with protection, or specific threshold values of assays which have been accepted or proposed as differentiating susceptible from protected individuals. We also use the term ‘protective threshold’ to refer to an assay value for the correlate that distinguishes protected and unprotected individuals when the relationship between the correlate and protection can be reliably and usefully summarized with a single threshold value. However, individual variability means that at any threshold value some above will be susceptible and some below protected, and ‘protective threshold’ is not intended to imply any particular level of protection, and specifically is not intended to imply complete protection or ‘sterile immunity’. ‘Assay value’ and ‘titer’ are used interchangeably, according to context. A general opinion is emerging that improvement in statistical methods is needed [10,11] for identifying correlates of protection, but opinions vary on the appropriate statistical methodology. Methods and study designs have varied historically and across disease areas resulting in different standards of data quality and statistical methods to establish correlates of protection and their threshold values.

For older vaccines, the protective immunological thresholds have often been determined based on observational data, which was sometimes conveniently available or opportunistic. For example, Björkholm et al. measured diphtheria antitoxin titers in 44 individuals admitted to hospital during a diphtheria epidemic among alcoholics in Sweden and observed that 7 of 10 patients who had diphtheria antitoxin titers < 0.01 IU/ml died or showed neurological complications, whereas 33 out of 34 diphtheria carriers with antitoxin titers ≥ 0.16 IU/ml remained symptom-free [12]. Further in vitro studies suggested that titers between 0.01 and 0.09 IU/ml may be regarded as giving basic immunity, whereas a higher titer of 0.1 IU/ml was considered fully protective [13].

When an outbreak of measles occurred among students in a dormitory at Boston University, Chen et al. obtained permission to assay samples of blood donations made shortly before the start of the outbreak and compared their antibody concentrations with the occurrences of measles [14]. Of 9 donors with detectable pre-exposure plaque reduction neutralization titer less than or equal to 120, 8 met the clinical criteria for measles compared with none of 71 with pre-exposure titers greater than 120. Similarly, Neumann collected sera from 238 high school students on Prince Edward Island before a measles epidemic sweeping the rest of Canada reached the island to compare infection rates by titer [15].

An early study by Goldschneider et al. established a protective threshold for meningococcal C disease based on serum bactericidal assay [16]. American army recruits provided blood samples for assaying at the start of basic training, and disease occurred in only 1% of individuals who had titers greater than 4 of SBA at recruitment compared to 22% of those who had less than 4. This was further confirmed by a population study that demonstrated an inverse relationship between disease incidence and the presence of SBA titers.

These early studies and others [17] selected protective thresholds based on inspection of disease rates observed in discrete intervals of assay values with confidence limits never reported. Siber provides an in-depth discussion of this approach [18] and introduces the idea of titer-specific degrees of protection.

For newer vaccines, clinical trials or observational studies specifically incorporate immunological data collection to identify potential thresholds, and statistical approaches have accordingly been developed for this purpose. For instance, in the Chang-Kohberger method data from three double-blind controlled trials in Northern Californian, American Indian and South African infants were pooled in a meta-analysis to derive a protective threshold of 0.35 μg/ml for anticapsular antibodies for a 7-valent pneumococcal conjugate vaccine against invasive pneumococcal disease [19,20]. The statistical method equates relative risk of invasive pneumococcal disease between vaccine and control groups to the relative risk of having antibody concentration below the protective threshold, and the protective threshold is then found from cumulative distribution curves of the antibody concentrations of the vaccinated group and the control groups. The threshold has been endorsed by a WHO Working Group and has subsequently been used to develop and license a newer generation 13-valent vaccine [21].

It was essentially this same method that was employed by Andrews et al. to derive a threshold for a correlate of protection following meningococcal C vaccination [22]. The two modern examples for pneumococcal and meningococcal C vaccines that employed the Chang-Kohberger method, however, required an estimate of vaccine efficacy based on a clinical endpoint before the method could be used.

Few other statistical methods exist for identifying a threshold. The idea of estimating separate disease probabilities *a* and *b* below and above a threshold has been proposed by Siber et al. but no actual model was developed to estimate the threshold [20].

Other statistical approaches have focused on continuous models, which do not explicitly model a threshold. Logistic regression has frequently been used [23-28]; other continuous models have included proportional hazards [29] and Bayesian generalized linear models [30]. Chan compared Weibull, log-normal, log-logistic and piecewise exponential models applied to varicella data [31]. A limitation of such models is that they cannot separate exposure to disease from protection against disease given exposure, the latter being the relationship of interest. A scaled logit model which separates exposure and protection where protection is a continuous function of assay value has been proposed [32]. The scaled logit model was illustrated with data from the German pertussis efficacy trial data [27] and has been used to describe the relationship between influenza assay titers and protection against influenza [33-35]. However, these approaches do not explicitly allow identification of a single threshold value.

Thus despite the fundamental reliance on thresholds in vaccine science and immunization policy, previous statistical models have not specifically incorporated a threshold parameter for estimation or testing. In this paper, we propose a statistical approach based on the suggestion in Siber et al. [20] for estimating and testing the threshold of an immunologic correlate by incorporating a threshold parameter, which is estimable by profile likelihood or least squares methods and can be tested based on a modified likelihood approach. The model does not require prior vaccination history to estimate the threshold and is therefore applicable to observational as well as randomized trial data. In addition to the threshold parameter the model contains two parameters for constant but different infection probabilities below and above the threshold and can be viewed as a step-shaped function where the step corresponds to the threshold. The model will be referred to as the a:b model.

## Methods

### Model specification and fitting

For subjects *i* = 1,…,*n*, let *t*_*i*_ represent the immunological assay value for subject *i* (typically immunological assay values are log-transformed before making calculations). Let *Y*_*i*_ = 1 represent the event that subject *i* subsequently develops disease, and *Y*_*i*_ = 0 the event that they do not and *τ* represent a threshold differentiating susceptible from protected individuals. Then the model is given by

PYi=1=a×1ti<τ+b×1ti>τPYi=0=1-a×1ti<τ-b×1ti>τ

where *a*, *b* represent the probability of disease below and above the threshold respectively and 1(·) takes the value 1 when its argument in parenthesis is true or 0 otherwise. Since the assay values *t*_*i*_ are discrete observations of a continuous variable, and the likelihood and residual sum of squares are each constant at any value of *τ* falling between a pair of adjacent observed discrete assay values, a reasonable choice for the candidate values of *τ* are the geometric means of adjacent pairs of ordered observed assay values (i.e. the arithmetic mean of log-transformed assay values). The log of the likelihood for the model is given by

$$\begin{array}{ccc} \;l\left(a,b,\tau \right) & \;= & \sum_{i=1}^{n}y_{i}\log\left[\alpha \times 1\left(t_{i}<\tau \right)+b\times 1\left(t_{1}>\tau \right)\right]\\ & & +\left(1-y_{i}\right)\log\left[1-\alpha \times 1\left(t_{i}<\tau \right)-b\times 1\left(t_{i}>\tau \right)\right] \end{array}$$

To fit the models, closed form expressions may be derived by maximum likelihood or least squares for estimators of the parameters *a, b* but not for *τ.* The estimators for *a, b* remain as functions of *τ*. Following the profile likelihood or least squares approach, the optimal value of *τ* may be found by proceeding through the candidate values, estimating the other parameters and the likelihood or sum of squared errors at each value. The value of *τ* that maximizes the likelihood or minimizes the sum of squares is the estimate for *τ*. The derivation of the least squares and maximum likelihood estimators of *a, b* is shown in the Additional file 1.

A previous method which seeks to identify a cut point is the maximal chi-square proposed by Miller [36]. Here a continuous variable which is predictive of a clinical outcome is dichotomized using a cut point with cases and non-cases displayed in a 2×2 table. The optimal cut point corresponds to the maximal chi-square associated with the 2×2 table. It can be shown that the estimated threshold *τ* selected by least squares in the a:b model corresponds to the optimal cut point obtained via the maximal chi-square method; a proof is given in the Additional file 1.

### Testing for the existence of a threshold

Note that in the absence of a threshold the model reduces to a constant probability of infection independent of assay value. Thus to test for the existence of a threshold, the likelihood of the a:b model including the threshold *τ* and different infection probabilities *a, b* below and above the threshold is compared to the likelihood of a model without a threshold but a constant infection probability *a*’ for all assay values. The test statistic is the difference of minus 2 times the likelihood of the models:

$$D=-2l\left(a,b,\tau \right)+2l\left(a^{\prime }\right)$$

However, the additional requirement *a* > *b* is imposed by requiring *D = 0* when *a* < *b* so the modified test statistic is

$$\begin{array}{l} D'=-2l\left(a,b,\tau \right)+2l\left(a'\right)\mathrm{for}\;a>b\\ D'=0\;\mathrm{for}\;a<b \end{array}$$

Simulations performed under the null hypothesis of no existence of threshold showed that under this hypothesis the distribution of *D*’ may be approximated by a chi-squared distribution with 3 degrees of freedom; thus *D*’ may be compared to a chi-squared distribution with 3 degrees of freedom for testing the null hypothesis of no threshold. The test is an unconditional significance test of the step function represented by *τ*, *a*, *b* compared to a constant probability of infection.

### Confidence interval for the threshold value

Confidence intervals for the threshold value may be calculated by non-parametric bootstrapping following standard methods [37]. Datasets were resampled 5000 times with replacement, and the lower and upper limits of the 95% confidence interval for the threshold were based on the 2.5 and the 97.5 percentiles of the estimates of *τ* from each resampling.

### Goodness-of-fit

Residuals defined by the differences between the observed dichotomous outcomes and the modeled probability of disease as in the a:b model are not normally distributed and hence goodness-of-fit methods relying on normality are inappropriate. Although Pearson and Chi-squared deviance residuals may be used for dichotomous outcomes, when the number of discrete values of the model predictors is large, such as for a continuous predictor like titers, their distributions are not well approximated by chi-squared distributions since the degrees of freedom increases with the number of discrete values. In such circumstances Hosmer and Lemeshow propose an approach in which the observed predictors are grouped into 10 groups defined by the deciles of the ordered predictors, and goodness-of-fit is estimated by the squared difference between observed and predicted infection rates in each group [38].

When applied to the a:b model, the goodness-of-fit test statistic is

$$C=\sum_{g=1}^{10}\;\frac{{\left(y_{.g}-m_{g}{\hat{\pi }}_{g}\right)}^{2}}{m_{g}{\hat{\pi }}_{g}\left(1-{\hat{\pi }}_{g}\right)}$$

where *g* indexes groups 1,…,10, *y*_*.g*_ is the observed number of cases in group *g*, *m*_*g*_ is the number of subjects in group *g*, and ${\hat{\pi }}_{g}$ is the predicted disease probability in the group, i.e. $\hat{a}$ or $\hat{b}$ (or a weighted average if the group includes the threshold). Simulations show *C* to follow a chi-squared distribution with 8 degrees of freedom when the model is true, so the goodness-of-fit may be quantified by the probability in the upper tail of this distribution. The test assesses whether the step function represented by the a:b model is an appropriate representation of infection or whether another relationship such as a gradual one between titer and infection might be more likely than a stepped relationship.

### Relative risk

The relative risk of disease above and below the threshold may be a more readily interpretable measure of the relevance of a fitted threshold. Note that relative risk is not suitable as a criteria for selecting a value of *τ*, since for different candidate values for *τ* the relative risk declines from approximately 0.5 at low assay values to near 0 at high values. However, having selected *τ*, the relative risk quantifies the difference between those above the threshold and those below in terms of the outcome of interest, namely probability of disease. The relative risk is estimated by $\hat{b}/\hat{a}\;$. An approximate 95% confidence interval for the relative risk (conditional on the estimated value of *τ*) can be obtained by parametric bootstrapping.

SAS statistical software was used for all analysis.

### Datasets

The a:b model was applied to 15 datasets from four studies. Briefly the datasets are:

•German pertussis datasets: eight assays for IgG or IgA antibodies against pertussis toxin (PT), pertactin (PRN), filamentous hemagluttinin (FHA) and fimbriae (FIM) and occurrence of 44 cases of disease in 1994 subjects from a sub-study of a pertussis vaccine efficacy trial conducted in Germany between 1991 and 1994 [27]. IgG antibodies are a humoral immune response whereas IgA antibodies are responses at mucosal sites.

•Piedra/respiratory syncytial virus (RSV) datasets: assays for antibody to RSV/A and RSV/B among subjects presenting with acute respiratory symptoms at a hospital in Texas, and subsequent disease confirmation in 34 of 175 subjects [26].

•White/varicella dataset: varicella glycoprotein assay for children vaccinated with varicella vaccine in clinical trials conducted between 1987 and 1989, and disease occurrence in 79 of 3459 subjects in 12 months of follow up [17].

•Swedish pertussis datasets: four assays (IgG antibodies for PT, PRN, FHA, FIM) from subjects exposed to pertussis by another household member and the subsequent development of disease in 92 of 209 subjects, from a sub-study of a vaccine efficacy trial conducted in Sweden between 1992 and 1995 [28].

## Results

### Threshold estimates, statistical significance and confidence intervals

**Figure 1:**
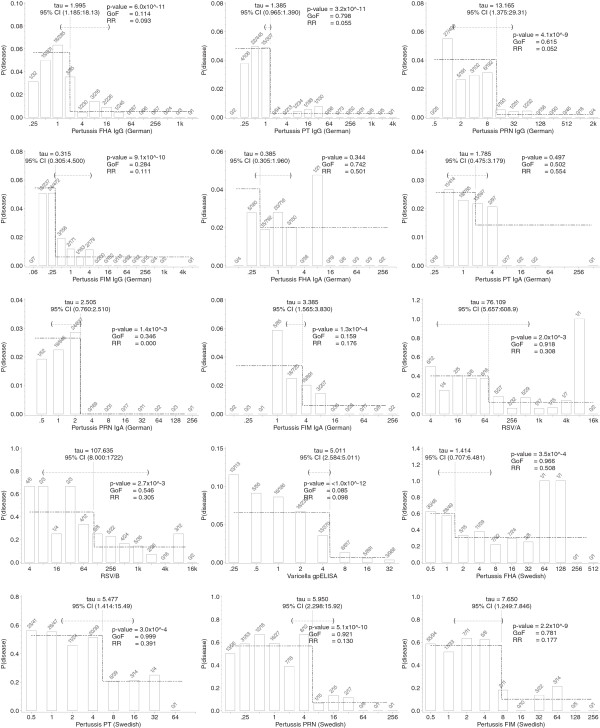
**Illustration of fitted a:b model for the 15 datasets.** Threshold values and 95% CIs for *τ* are superposed on the observed data showing the infection rates by titer value. The numbers above each bar show the number of cases of disease and the number of subjects at each binned assay value. Thresholds illustrated are those obtained by profile likelihood estimation. P-values refer to the modified likelihood ratio test with small values indicating statistical significance. GoF refers to the p-value of the goodness-of-fit test with small values implying a poor fit of the model to the data. RR is relative risk of infection above and below the threshold.

Figure 1 illustrates the application of the a:b model to the 15 datasets where the model fit showing *τ*, *a*, *b* is superposed on the observed data showing the infection rates by titer value. Table 1 lists the values of each threshold estimated by profile likelihood or least squares, their 95% confidence intervals (CIs) obtained by bootstrap, p-values for test for threshold and goodness-of-fit, and relative risk with CIs.

**Table 1:** **Correlate of protection threshold values**$\hat{\tau }$**estimated by a:b model and evaluation criteria for 15 immunological datasets on pertussis, RSV and varicella**

**Dataset (cases of disease: subjects)**	$\hat{\tau }$**by profile likelihood (95% CI)**	$\hat{\tau }$**by least-squares (95% CI)**	**p-value, test for threshold**^**†**^	**p-value, goodness-of-fit**^**†**^	**Relative risk**^**† **^**(95% CI)**
German pertussis FHA IgG (44:1988)	1.995 (1.185;18.13)	1.995 (0.990;2.025)	6.0×10^–11^	0.114	0.093 (0.030;0.183)
German pertussis PT IgG (44:1987)	1.385 (0.965;1.390)	1.385 (0.755;1.390)	3.2×10^–11^	0.798	0.055 (0.000;0.133)
German pertussis PRN IgG (44:1992)	13.165 (1.375;29.31)	7.665 (0.855;13.17)	4.1×10^–9^	0.615	0.052 (0.000;0.141)
German pertussis FIM IgG (44:1986)	0.315 (0.305;4.500)	0.315 (0.215;0.540)	9.1×10^–10^	0.284	0.111 (0.040;0.216)
German pertussis FHA IgA (44:1932)	0.385 (0.305;1.960)	0.385 (0.315;1.960)	0.344	0.742	0.501 (0.267;1.237)
German pertussis PT IgA (44:1933)	1.785 (0.475;3.179)	1.785 (0.415;4.064)	0.497	0.502	0.554 (0.181;1.120)
German pertussis PRN IgA (44:1968)	2.505 (0.760;2.510)	2.505 (0.485;2.510)	1.4×10^–3^	0.346	0.000 ( - ; - )
German pertussis FIM IgA (44:1994)	3.385 (1.565;3.830)	1.575 (1.030;3.825)	1.3×10^–4^	0.159	0.176 (0.037;0.375)
Piedra RSV/A (34:175)	76.109 (5.657;608.9)	76.109 (4.757;215.3)	2.0×10^–3^	0.918	0.308 (0.163;0.544)
Piedra RSV/B (34:175)	107.635 (8.000;1722)	107.635 (5.657;861.1)	2.7×10^–3^	0.546	0.305 (0.169;0.548)
White/Varicella (79:3459)	5.011 (2.584;5.011)	2.584 (1.311;5.011)	<1.0×10^–12^	0.085	0.098 (0.053;0.163)
Swedish pertussis FHA IgG (92:209)	1.414 (0.707;6.481)	1.414 (0.707;6.481)	3.5×10^–4^	0.966	0.508 (0.358;0.687)
Swedish pertussis PT IgG (92:209)	5.477 (1.414;15.49)	5.477 (1.414;10.49)	3.0×10^–4^	0.999	0.391 (0.199;0.614)
Swedish pertussis PRN IgG (92:209)	5.950 (2.298;15.92)	5.950 (1.497;6.380)	5.1×10^–10^	0.921	0.130 (0.030;0.270)
Swedish pertussis FIM IgG (92:209)	7.650 (1.249;7.846)	4.225 (1.249;7.846)	2.2×10^–09^	0.781	0.177 (0.056;0.325)

^†^for $\hat{\tau }$ found by profile likelihood.

For 12 of 15 datasets least squares and profile likelihood estimates of *τ* were the same while in the other 3 datasets (German pertussis PRN IgG, German pertussis FIM IgA, White/varicella) the least squares estimate was lower than the profile likelihood estimate.

Thirteen of 15 thresholds found by the model were highly statistically significant by the modified likelihood ratio test with p-values <0.01, while two German pertussis datasets for FHA IgA and PT IgA were not significant at the 0.05 level.

There was considerable variability in the widths of the 95% confidence intervals when considered relative to the range of the titers (Figure 1). In one instance, the German pertussis PT IgG data, the confidence interval was notably narrow; in the cases of the RSV/A and RSV/B datasets, the confidence intervals spanned a large proportion of the range of the titers. When fitted by profile likelihood, the point estimate of the threshold for German Pertussis PT IgG, PRN IgA, FIM IgA, White/varicella and Swedish Pertussis FIM datasets was close to the upper limit of the 95% CI and close to the lower limit for the German pertussis FIM IgG dataset. A similar pattern was seen for some datasets when fitted by least squares.

The upper and lower limits of the confidence intervals found by profile likelihood were often found to be greater than by least squares.

### Goodness-of-fit

Using the ad-hoc criterion that a goodness-of-fit p-value less than 0.20 represents a poor fit to the data, we found that the a:b model did not fit well to three datasets: White/varicella, German pertussis FHA IgG and German pertussis FIM IgA. Visual inspection of the plots in Figure 1 would suggest that protection against varicella follows a gradually increasing protection rate by titer value rather than a stepwise relationship, explaining the poor fit in this case. The German pertussis FHA IgG and FIM IgA appear to follow a similar gradual protection relationship. Another correlate of protection which may not be well described by the a:b model based on visual inspection of plots is RSV/B, but this was associated with a goodness-of-fit p-value of 0.546. Apart from RSV/B, all other datasets which were associated with goodness-of-fit p-values >0.20 could be visually confirmed to fit the stepwise shape of the a:b model.

### Relative risk

The relative risk estimate is dependent on the estimated threshold, and offers an interpretation which is more familiar to the epidemiologist. The relative risk of disease above the threshold compared to below ranged from 0 to 0.554 among the fifteen datasets. Except for 3 relative risks with values near 0.5 and one near 0.4, all other relative risks took values near 0.3 or less implying protection of 70% or better. Thus, in most cases, the estimated threshold corresponds with the notion of an absolute correlate to offer a high degree of protection.

## Discussion

Despite the central importance of threshold values in vaccines research and immunization policy, only the Chang-Kohberger method [19,20] has been previously proposed to estimate thresholds from assay values and disease occurrence data, but its estimation requires information on vaccinated and unvaccinated groups. The a:b method provides a reliable, readily applicable method for finding a threshold for paired data of the form {*y*_*i*_,*t*_*i*_} for which previous models and associated statistical testing were limited. The a:b model provides the same estimate as the maximal chi-square method [35] when least squares estimation is used.

The statistical criteria available for the evaluation of a threshold estimated by the a:b model are confidence interval width and location, goodness of fit, significance testing and relative risk. A number of factors are likely to influence the width of confidence intervals, including the presence of a clear, high step in the data and the number of subjects and cases of disease in the dataset. Further, bootstrap confidence intervals based on the candidate values of tau are affected by the density of distinct observed assay values in the region of the threshold. This is a data limitation arising from the assay technique which generates discrete rather than continuous titer values, with lower densities (fewer distinct assay values) tending to produce wider confidence intervals and higher densities allowing the possibility of smaller confidence intervals. The location of threshold point estimates and upper and lower confidence limits in some datasets suggested that profile likelihood estimates may be higher and therefore more conservative, requiring higher antibody titers to be achieved to conclude protection, compared to least squares estimates.

Goodness-of-fit p-value in some instances was clearly consistent with the bar plots of the binned data while in other cases this was less so, possibly due to discreteness in the data resulting from small numbers of cases of disease. Visual inspection of graphical representations of the data might routinely supplement statistical assessments.

Because the estimated threshold itself does not imply the degree of protection, relative risk aids in its interpretation. If a threshold is to separate susceptible from protected individuals, relative risk may be seen as a measure of the degree of protection and can be employed as one of the criteria for assessing the relevance of an estimated threshold in addition to the p-value from the test for significance. For example, the Swedish pertussis FHA IgG result produced a p-value of 3.5×10^-4^ but a relative risk of 0.508, implying around 50% reduction in risk, which may question the acceptability of the threshold as higher protection is generally expected in vaccine preventable disease.

Ideally, all assessment criteria would provide consistent results in support of a threshold. However, instances were noted where other conclusions might be warranted even though some statistical assessments were promising. For example, for the White/varicella data, there is a small confidence interval for the threshold, the p-value for the threshold is highly significant and the relative risk acceptable (close to 0.1) but the goodness-of-fit is poor (p = 0.085). It was found that that this data is better fitted by a continuous scaled-logit model (p for goodness-of-fit = 0.999), suggesting that a relative rather than absolute threshold may be appropriate.

The threshold in the a:b model is the titre value that best separates the sample of patients into two groups with different but constant infection rates, but this does not require the ‘protected’ group to have a specified low probability of infection. It is therefore possible that the protected group defined by the estimated threshold has a high probability of infection, like 20% in the pertussis PT IgG example, which could be deemed to be unacceptably high if one’s definition of a threshold requires low risk of infection. Therefore, an additional criterion that sets a maximally acceptable probability of infection amongst the protected group could be considered in addition to statistical tests when evaluating thresholds.

Although definitions of thresholds may differ, it is encouraging to note that others’ published estimates of thresholds for these same datasets are not dissimilar to estimates from the a:b model, suggesting consistency with others’ notion of an acceptable threshold. For instance, a previous analysis of the White/varicella data identified a gp ELISA titer of 5 U/mL to indicate protection, which is now reported to be an ‘approximate correlate of protection’ for varicella vaccines [39]. The estimate was consistent with our profile likelihood estimate of the threshold of 5.011 (95% CI; 2.584; 5.011). For the Swedish pertussis data, a putative threshold value of 5 units/mL for PRN, FIM and PT were found to be associated with high protection [28]; subjects having all three had even higher protection. However, while the authors applied the same putative threshold to all 3 pertussis components, we estimated different values for each: 5.477 (95% CI; 1.414;15.49) for PT, 5.950 (95% CI; 2.298;15.92) for PRN and 7.650 (95% CI; 1.249;7.846) for FIM. For the German pertussis data, a regression tree approach found that a threshold value of 7 units/mL for PRN IgG was most predictive of protection [23]. We estimated a threshold of 13.165 (95% CI; 1.375;29.31) with profile likelihood and 7.665 (95% CI; 0.855;13.17) using least squares. Amongst the subset of subjects achieving 7 units/mL for PRN, those who had 66 units/mL of PT IgG had even greater protection. Our estimated threshold for PT IgG using profile likelihood was 1.385 (95% CI; 0.965;1.390), but this figure is not comparable to the previous figure of 66 unit/mL which should be interpreted as a conditional threshold given that protective PRN levels are achieved.

Because the a:b model assumes constant rates of infection on each side of the threshold, which may be a strong assumption, we considered in supplementary analyses more flexible models which allowed linear, quadratic or logistic relationships on either side of the threshold. However, these models did not produce fits corresponding with the expectations of a correlate of protection. For instance, a step-down of infection rate at the threshold value and non-increasing rates of infection on either side of the threshold were not always observed. The a:b model was always consistent with these expectations. In addition, visual examination of the profile likelihood for these other models did not show sharp peaks corresponding to the optimal threshold value, and were associated with wider confidence intervals resulting in greater uncertainty of the threshold value. In general these more flexible models could not be relied upon to consistently find a threshold which could be said to differentiate protected from susceptible individuals.

The a:b model presented here does not require vaccination information to estimate a threshold. While this is an advantage, it is also a weakness given that the a:b model can provide only the first level of information in the hierarchy of evidence to demonstrate a statistical correlate of vaccine efficacy in the framework described by Qin et al. [7]. To provide a higher level of evidence, the a:b model could be developed to include a vaccination parameter and an associated test. Also, further development could allow for multiple co-correlates in which two or three threshold values are estimated simultaneously. This could have application to diseases like pertussis where more than one antigen is necessary for the fullest protection or for new vaccines that protect against multiple serotypes of a disease, such as pneumococcal infection or dengue. Further research might also compare different statistical models for correlates of protection – the a:b model, the method of Chang and Kohberger [19-21], the scaled logit model [32-35], a linear trend model and logistic regression – and the conclusions reached by each for levels of protection.

In order to investigate correlates of protection and thresholds, there are also clinical and immunological considerations. A correlate must include a clearly defined clinical endpoint, whether protection is afforded against infection, disease, severe disease, infectiousness, carriage or other condition. For instance, it is thought that protection against pneumococcal infection requires progressively lower thresholds for protection against pneumococcal carriage, otitis media, pneumonia and invasive pneumococcal infection [40]. Similarly, standardized laboratory assays and tests for disease case confirmation are also needed but not always feasible, which can potentially introduce bias in laboratory confirmed disease cases in some studies. An assay must first be selected by immunologists and validated according to immunological criteria – sensitivity, specificity, reliability, and freedom from inter-technician variability. It may be of interest to know whether the specific immune response measured by the assay is responsible for protection; statistical methods for causal inference have recently been developed allowing an assay to be selected which has been shown to be causally associated with protection [41,42]. Other considerations include: host factors in which the immune system changes throughout life implying different immune response by age, temporal immunological factors such as timing of measurement and kinetics of the immune response, and population factors given that observed thresholds may not be universally applicable to all settings. Thus, once a correlate of protection or threshold is proposed, further discussions with stakeholders are necessary to cover these disease-specific considerations that the statistical methods alone cannot address.

A final practical requirement is that datasets to identify immunological correlates of protection are essential. Vaccine efficacy trials provide a clear opportunity to collect data on the relationship between assay values for candidate correlates of protection and disease occurrence; however, they are often sized inadequately to yield convincing conclusions on correlates of protection. Typically trials are designed to capture 40–100 cases of disease to convincingly demonstrate adequate vaccine efficacy against placebo [43-45], but such trials are generally underpowered for assessing correlates of protection. Incorporation of a correlate of protection objective in clinical trials can incur substantial expense to the trial as it would require additional bleeds in subjects after they receive vaccine or placebo to observe their assay values and before any significant number of disease cases occur. Furthermore, more refined titer measures (i.e. less discrete data) would require more serial dilutions and greater blood volumes.

## Conclusions

The a:b model together with the evaluation criteria proposed provide a much-needed set of methods for the estimation and assessment of thresholds values of immunological correlates of protection.

## Competing interests

The authors declare that they have no competing interests.

## Authors’ contributions

All authors contributed to the formulation of the research question, made methodological suggestions for consideration and evaluation by the group, and contributed to the interpretation of the results. XC, FB and AD performed the statistical calculations and KD and AD drafted the manuscript. All authors read and approved the final version.

## Pre-publication history

The pre-publication history for this paper can be accessed here:

http://www.biomedcentral.com/1471-2288/13/29/prepub

## Supplementary Material

Additional file 1: **b model and on equivalence of Miller’s maximal chi-square and least-square estimates of a:b model.** (DOC 61 kb) Click here for file
