# Erratum to: A threshold method for immunological correlates of protection

**DOI:** 10.1186/s12874-016-0279-z

**Published:** 2017-02-02

**Authors:** Xuan Chen, Fabrice Bailleux, Kamal Desai, Li Qin, Andrew J. Dunning

**Affiliations:** 1 Sanofi Pasteur, Beijing, China; 2 Sanofi Pasteur, Marcy L’Etoile, France; 3 United Biosource Corporation, London, UK; 4 Statistical Center for HIV/AIDS Research and Prevention, Fred Hutchinson Cancer Research Center, Seattle, WA USA; 5 Present address: Amazon.com, Inc, Seattle, WA USA; 6 Sanofi Pasteur, Swiftwater, PA USA

## Erratum

After publication of the original article [1], it came to the authors’ attention that there was an error in the **Testing for the existence of a threshold** sub-section of the Methods.

Two formulae originally published in this sub-section were incorrect; the correct expressions are the negatives of the expressions published. The formulae should have appeared as follows:

$$ \mathrm{D} = 2\mathrm{l}\left(\mathrm{a},\mathrm{b},\uptau \right)\ \hbox{--}\ 2\mathrm{l}\left({\mathrm{a}}^{'}\right) $$

and

$$ \mathrm{D}' = 2\mathrm{l}\left(\mathrm{a},\mathrm{b},\uptau \right)\ \hbox{--}\ 2\mathrm{l}\left(\mathrm{a}'\right)\ \mathrm{f}\mathrm{o}\mathrm{r}\ \mathrm{a} > \mathrm{b}. $$
