# Supplementary material for: A threshold method for immunological correlates of protection
Source: BMC Med Res Methodol. 2013 Mar 1;13:29. doi: 10.1186/1471-2288-13-29 (PMC3639076; doi:10.1186/1471-2288-13-29)
Supplement: Additional file 1: — b model and on equivalence of Miller’s maximal chi-square and least-square estimates of a:b model. (DOC 61 kb) [file 1471-2288-13-29-S1.doc]

# Estimating equations

The derivation of the estimating equations for the a:b model is shown. The notation is defined in section 3.1. Stated as a probability model, the model is

$$\mathrm{P}\left(Y_{i}=1\right)=a\times1\left(t_{i}<\tau\right)+b\times1\left(t_{i}>\tau\right)$$

The log-likelihood is

$\mathrm{l}\left(a,b,\tau\right)= \sum_{i} y_{i}\log\left[a\times1\left(t_{i}<\tau\right)+b\times1\left(t_{i}>\tau\right)\right]+\left(1-y_{i}\right)\log\left[1-a\times1\left(t_{i}<\tau\right)-b\times1\left(t_{i}>\tau\right)\right]$ .

Setting the first derivative with respect to *a* to 0 yields the estimating equation

$$\sum y_{i}-a\sum1= 0$$

except when *a* = 0 or 1, sums being taken over {*i*:*t_i_*<*τ*}; thus

$\hat{a}=\frac{\sum y_{i}}{\sum1}$.

The estimator for *b* is similar, sums being taken over {*i*:*t_i_*>*τ*}. The second derivative of the log-likelihood,$\frac{d^{2}l}{da^{2}}$, may be seen to be always negative, indicating that the solution maximizes the likelihood; similarly for *b*. The first derivative of the log-likelihood with respect to *τ* is undefined when *τ* equals an assay value, and zero otherwise. There is no closed form expression for the estimator of *τ* and so it is estimated by maximizing the profile likelihood, i.e. calculating $\hat{a}\text{ and }\hat{b}$and hence the likelihood at each candidate value of *τ* and selecting the value maximizing the likelihood.

As a least squares model the model is

$$y_{i}=a\times1\left(t_{i}<\tau\right)+b\times1\left(t_{i}>\tau\right)+\varepsilon_{i}$$

where *ε_i_* is the error for subject *i*. The sum of squared errors is

$$\sum\varepsilon_{i}^{2}= \sum y_{i}^{2}+a^{2}\times1\left(t_{i}<\tau\right)+b^{2}\times1\left(t_{i}>\tau\right)-2y_{i}a\times1\left(t_{i}<\tau\right)-2y_{i}b\times1\left(t_{i}>\tau\right)$$

Setting the first derivative with respect to *a* to 0 yields the same estimating equation as at above. The second derivative of the sum of squared errors,$\frac{d^{2}\sum\varepsilon_{i}^{2}}{da^{2}}$, may also be seen to be always positive, indicating that the solution minimizes the sum of squared errors; similarly for *b*.

The first derivative of the sum of squared errors with respect to *τ* is undefined when *τ* equals an assay value, and zero otherwise. There is no closed form expression for the estimator of *τ* and so it is estimated by selecting from among the candidate values of *τ* the value that minimizes the sum of squared errors, using $\hat{a}\text{ and }\hat{b}$ for *a* and *b* respectively, calculated at each candidate value.

# Equivalence of least-squares estimate of a:b model with Miller’s maximal chi-square

The maximal chi-square selects the value of *τ* yielding the greatest value of the *χ*^2^ statistic. Using the following notation for the number of subjects above and below the threshold *τ* and the number with and without disease

|  | Disease status | |  |
| --- | --- | --- | --- |
|  | *y_i_*=0 | *y_i_*=1 | Total |
| *t_i_*<*τ* | *n*_00_ | *n*_01_ | *n*_0⋅_ |
| *t_i_*>*τ* | *n*_10_ | *n*_11_ | *n*_1⋅_ |
| Total | *n*_⋅0_ | *n*_⋅1_ | *n* |

the *χ*^2^ statistic is

$$\chi^{2}=\frac{\left(n_{00}-\frac{n_{0.}n_{.0}}{n}\right)^{2}}{\frac{n_{0.}n_{.0}}{n}}+\frac{\left(n_{01}-\frac{n_{0.}n_{.1}}{n}\right)^{2}}{\frac{n_{0.}n_{.1}}{n}}+\frac{\left(n_{10}-\frac{n_{1.}n_{.0}}{n}\right)^{2}}{\frac{n_{1.}n_{.0}}{n}}+\frac{\left(n_{11}-\frac{n_{1.}n_{.1}}{n}\right)^{2}}{\frac{n_{1.}n_{.1}}{n}}$$

As a least squares model the a:b model is *y_i_* = *a*×1(*t_i_*<*τ*) + *b*×1(*t_i_*>*τ*) + *ε_i_*, the estimators of *a* and *b* are

$\hat{a}=\frac{\sum y_{i}\times1\left(t_{i}<\tau\right)}{\sum1\left(t_{i}<\tau\right)}=\frac{n_{01}}{n_{0\cdot}}$ and $\hat{b}= \frac{\sum y_{i}\times1\left(t_{i}>\tau\right)}{\sum1\left(t_{i}>\tau\right)}=\frac{n_{11}}{n_{1\cdot}}$

and the sum of squared errors is

$$\begin{matrix} \sum\varepsilon_{i}^{2}= \sum\left(y_{i}-\frac{n_{01}}{n_{0.}}\times1\left(t_{i}<\tau\right)-\frac{n_{11}}{n_{1.}}\times1\left(t_{i}>\tau\right)\right)^{2} \\ = n_{.1}-\frac{n_{01}^{2}}{n_{0.}}-\frac{n_{11}^{2}}{n_{1.}} \end{matrix}$$

By substitution it may be seen that

$$\chi^{2}= \frac{n}{n_{.0}}\left(n-n_{.1}\right)-n \left(\frac{1}{n_{.1}}+\frac{1}{n_{.0}}\right)\times\sum\varepsilon_{i}^{2}$$

Thus, conditional on the marginal totals, the *χ*^2^ statistic is a strictly decreasing function of sum of squared errors, and the value of *τ* minimizing the sum of squared errors will maximize the value of the *χ*^2^ statistic.
